# Does diabetes mellitus affect the safety profile of valproic acid for the treatment of status epilepticus? A retrospective cohort study

**DOI:** 10.1186/s42466-022-00212-w

**Published:** 2022-10-24

**Authors:** Annekatrin Müller, Judith von Hofen-Hohloch, Carolin Awissus, Jens Przybilla, Achmed Mrestani, Joseph Classen

**Affiliations:** 1 Department of Neurology, Leipzig University Medical Center, Liebigstraße 20, 04103 Leipzig, Germany; 2 Institute for Medical Informatics, Statistics and Epidemiology, Leipzig University, Leipzig, Germany

**Keywords:** Status epilepticus, Valproic acid, Diabetes mellitus, Safety

## Abstract

**Background:**

In the treatment of status epilepticus less is known about the influence of comorbidities on the safety profile of anticonvulsive drugs. Especially patients with diabetes mellitus may be predisposed to certain adverse events that have been related to therapy with valproic acid. In this single-center retrospective cohort study we examined if the complications of the intravenous treatment with valproic acid is different in patients with or without diabetes.

**Methods:**

Patients who were treated for status epilepticus with intravenous valproic acid between 2008 and 2020 were identified. Primary endpoint was the discontinuation of therapy with valproic acid due to adverse events. Relevant secondary endpoints were the functional status at the time of discharge from hospital in comparison to the premorbid state and the in-hospital mortality. Both groups (patients with or without diabetes) were compared by Mann–Whitney U-Test or Pearson´s Chi^2^ test. To identify therapy with valproic acid as a risk factor of in-hospital mortality, a binary regression model was used.

**Results:**

During the study period 408 patients and 482 episodes of status epilepticus were treated with intravenous valproic acid. Group comparisons did not reveal a significant difference in the rates of discontinuation of therapy. A difference was found in the rate of thrombocytopenia (*p* = 0.015), which occurred more often in patients with diabetes. In total, 36 hypoglycemic episodes could be identified, two occurred spontaneously under intravenous valproic acid. After correction for potential confounders, continuous therapy with valproic acid could not be confirmed as an independent risk factor for in-hospital mortality (*p* = 0.079). In patients with diabetes, the proportion of patients with a good functional state, indicated by the modified Rankin Scale, was significantly lower in both times (premorbid: 55% vs. 69%, *p* = 0.008; at discharge: 22% vs. 36%, *p* = 0.004).

**Conclusions:**

Tolerability of the treatment with valproic acid was similar in patients with or without diabetes. Diabetes as a relevant comorbidity can signal a potentially increased risk of a poor outcome after status epilepticus.

*Trial registration*: The study was registered at the German Clinical Trials Register on 8 April 2022 (DRKS 00,027,836).

**Supplementary Information:**

The online version contains supplementary material available at 10.1186/s42466-022-00212-w.

## Background

Status epilepticus (SE) is after stroke the most frequent neurological emergency condition with case fatality rates of about 15% [1]. Previous research has focused on potential prognostic factors of SE, including age, etiologies and therapeutic regimes [2–4]. With regard to anticonvulsive drugs, efficacy and the general safety profile have been reported [e.g., 5]. However, to our knowledge, no analysis has been performed on the influence of individual comorbidities on the safety of specific anticonvulsive drugs.

Valproic acid (VPA) is an established therapy for the treatment of SE in Germany and many other European countries [6]. It is recommended as one of the second line drugs after failure of benzodiazepines. According to the most recent guidelines from Italy and Germany, additional second-line therapies for the treatment of SE include levetiracetam and (fos)phenytoin [7, 8]. Published data reveal a good safety profile of VPA even in critically ill patients [5, 9]. No safety signals have emerged from international published safety studies or other trials (reviewed in Trinka et al., 2014 [5]) including the prospective Established Status Epilepticus Treatment Trial [ESETT; 9, 10]. On the other hand, patients with certain comorbidities have not been considered separately. However, treatment with VPA is known to be associated with adverse events that may occur more frequently in specific risk populations.

Here, we asked whether the safety profile of intravenous (IV) VPA treatment of SE is modulated by the presence or absence of diabetes mellitus, particularly insulin-dependent diabetes mellitus (IDDM). Our question was motivated by the increasing frequency of diabetes in the general population [11]. Furthermore, VPA is associated with adverse events to which patients with diabetes are predisposed, such as disturbance of the glucose metabolism or pancreatic damage.

Therefore, the present retrospective study in a single-academic center cohort examined if, all other available factors considered, complications of IV treatment with VPA were different in patients with and without known concomitant diabetes. Outcome relevant issues included the in-hospital mortality and the modified Rankin Scale [mRS, 12] at the time of discharge from hospital.

## Methods/design

### Study design

The present study was conducted in a retrospective, non-interventional design (DRKS 00,027,836). It was approved by the local Ethics Committee (05/2021; 227/21-ek).

### Study population, clinical data and definitions

Adult patients (≥ 18 years) who were treated for SE between 01.01.2008 and 31.12.2020 at the Department of Neurology were identified through a review of medical records. Patients were admitted via the emergency department, or they were referred for emergency treatment from other hospitals. All patients with an indication for an ICU treatment and without known restrictions regarding intensive care therapy were treated at the neurological ICU. SE was defined clinically as epileptic convulsions lasting at least 5 min, or as repetitive occurrence of seizures without full recovery of consciousness in between. Patients with nonconvulsive SE were identified through the review of reports of routine electroencephalography. Only SE episodes treated with IV VPA (single infusion or repeated/ continuous infusions) were included. In general, treatment with IV VPA was administered in the following ways: (1) Single infusion with subsequent continuous application via perfusor; (2) single infusion followed by oral therapy; (3) repeated infusions with a maximum of five times per day; (4) Stop after single infusion.

For each SE episode, the comorbidity burden was scored with the original Charlson Comorbidity Index (CCI, 13). Parameters of the CCI were defined as followed: cerebrovascular diseases without severe residual symptoms, 1 point; hemiparesis that prevented walking, 2 points; dementia, defined as chronic cognitive impairment severe enough to interfere with the patient's daily life and activities, 1 point. All diseases newly diagnosed in the present hospital stay were considered in the CCI. For most analyses, patients were stratified into those with or without diabetes. Patients with a history of or newly diagnosed diabetes were assigned to the first group. Diabetes was defined as insulin-dependent if insulin was part of the premorbid treatment regimen and/or insulin was administered during the hospital stay and continuation of insulin therapy was recommended after hospital discharge. SE episodes were graduated with the Status Epilepticus Severity Score (STESS; range, 0–6). SE episodes were dichotomized into STESS of 3 or higher or STESS of less than three points as proposed in previous studies [2, 14, 15]. Etiologies potentially leading to death independently of SE, such as acute large vessel ischemic stroke, acute cerebral hemorrhage or acute central nervous system infection were recorded [16]. If VPA was discontinued in relation to adverse events, the records were evaluated for one or several of the following conditions diagnosed by the treating team: disturbance of consciousness (which could not be strictly related to an ictal or postictal state in the opinion of the medical staff), thrombocytopenia, parkinsonism, elevation of liver enzymes, bleeding, others. Thrombocytopenia was attributed to the therapy with VPA, if (1) platelet levels were normal before IV administration of VPA OR (2) there was an aggravation of a pre-existing thrombocytopenia under IV VPA and previous oral maintenance therapy with VPA AND platelet levels recovered after withdrawal from therapy. In addition, other complications of in-hospital treatment that may be attributed to VPA therapy were obtained from the hospital records: need for mechanical ventilation, bleedings that required intervention and/ or blood transfusion, episodes of hypoglycemia (single vs. recurrent, blood glucose level < 3.3 mmol/L) and severe pancreatic damage.

### Outcome definitions

Primary endpoint was the rate of discontinuation of the VPA therapy due to adverse events. Secondary endpoints were: need for mechanical ventilation, episodes of hypoglycemia, bleedings that require intervention, pancreatic damage, rate of in-hospital mortality associated with continuous or repeated VPA infusions, and the functional status at discharge from hospital as indicated by the mRS in comparison with the premorbid mRS. Primary and secondary endpoints were assessed in patients with and without diabetes. The outcome parameters (functional status at discharge from hospital and mortality) were also assessed in the total cohort.

### Statistical analysis

For statistical analysis, we considered the first SE episode from each patient which was treated with VPA. The study cohort was described by median and interquartile range for continuous and number (percent) for categorical characteristics. Accordingly, groups were compared by Mann–Whitney U- test. Categorical variables were compared using the Pearson´s Chi^2^ test or the Fisher´s exact test. To identify VPA therapy as a potential risk factor of in-hospital mortality, the binary logistic regression model was used. Variables included in the model were selected based on a preceding univariate analysis. Only variables showing a significant difference in univariate analysis were considered candidate covariates for the multivariate model.

In case of a significantly different occurrence of an adverse event, we corrected the result for age and sex also using a binary regression model. For the comparison of the premorbid functional state with the functional outcome at discharge, only patients in whom both mRS values were available were considered. Analysis was performed using the Wilcoxon test for paired samples. To investigate the interaction between group assignment (diabetic vs. non-diabetic, between subject factor) and the point in time of mRS determination (within subject factor), mixed ANOVA analysis was performed. The general significance level was set at α = 5% for two-tailed testing. Data preparation, descriptive statistics, correlation analysis and nonparametric tests were performed with IBM SPSS Statistics, version 27 and 28. Illustrations were created with Microsoft Power Point, SigmaPlot 13.0 and Adobe Illustrator 2020 24.0.1.

## Results

### Demographic and clinical characteristics of the study cohort

**Fig. 1:**
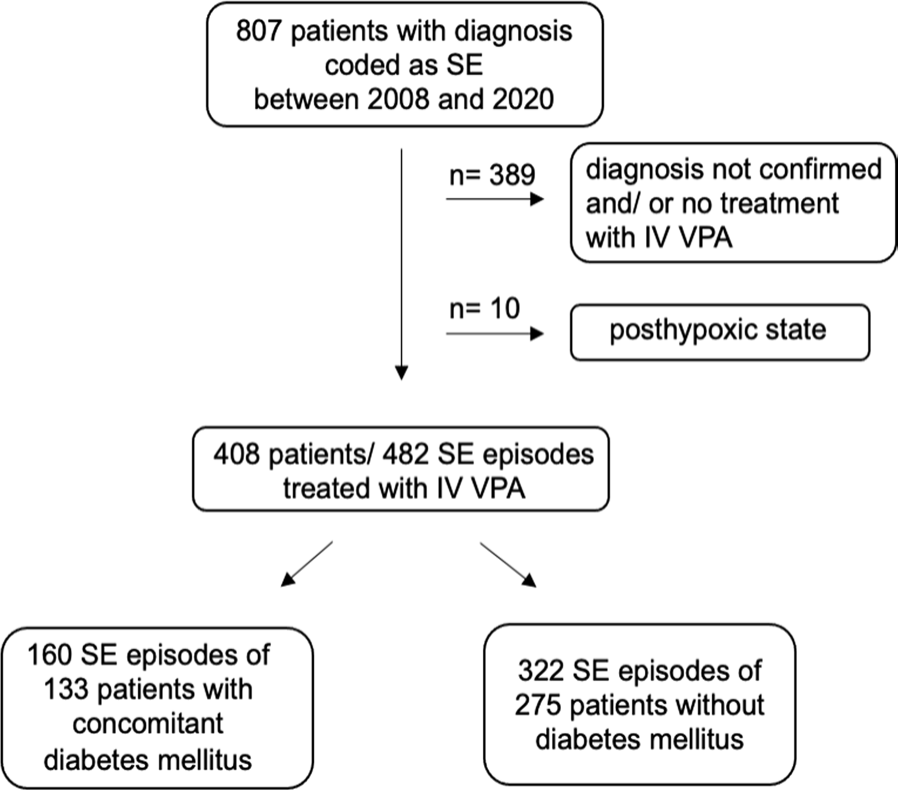
Flow chart of the study cohort. Abbreviations: SE, status epilepticus; IV, intravenous; VPA, valproic acid

We identified 807 patients who were treated in the Department of Neurology at Leipzig University Hospital between 2008 and 2020 and whose hospital records were coded as SE. In this group, 408 patients, in whom the diagnosis of SE was verified using the criteria outlined in “Methods”, received VPA via an IV route. A total of 482 episodes of SE treated with IV VPA were identified (Fig. 1). Concomitant diabetes was known in 133 patients including 55 patients with IDDM (13%). The median age of the total cohort was 73 years (62–81 years). The majority of patients (90%) were treated with VPA either continuously or with repeated infusions. Regarding dosage and time of start, no significant differences between the group of patients with or without diabetes could be found (Additional file 1).

The main clinical characteristics including relevant adverse events potentially related to VPA therapy are listed in Table 1.

**Table 1:** Characteristics of the total study cohort and both groups (patients with or without known diabetes)

Characteristics	Total cohort	Group, diabetes	Group, no diabetes	*p*-value
Patients	N = 408	N = 133	N = 275	
*Demographics*				
Age	73 (62–81)	75 (70–81)	71 (58–81)	**0.001**
Sex: m (male) f (female)	m 188 (46%), f 220 (54%)	m 51 (38%), f 82 (62%)	m 137 (50%), f 138 (50%)	**0.034**
*Comorbidities*				
Charlson Comorbidity Index	3 (1–4)	4 (3–6)	2 (1–3)	** < 0.001**
Insulin-dependent diabetes mellitus	55 (14%)	55 (42%)	0	** < 0.001**
Active abuse of alcohol*	41 (10%)	10 (8%)	31 (11%)	0.293
*Status epilepticus*				
STESS ≥ 3	272 (67%)	106 (80%)	166 (60%)	** < 0.001**
Worst seizure type: GCSE	171 (42%)	47 (35%)	124 (45%)	0.061
Worst seizure type: NCSE with coma*	12 (3%)	3 (2%)	9 (3%)	0.758
Consciousness: Stuporous or comatose	306 (75%)	99 (74%)	207 (75%)	0.855
History of seizures	202 (50%)	48 (36%)	154 (55%)	** < 0.001**
*Etiology of SE*				
Potentially fatal etiology	126 (31%)	47 (35%)	79 (29%)	0.175
*Treatment with VPA*				
VPA single infusion*	42 (10%)	10 (8%)	32 (12%)	0.227
VPA repeated or continuous infusion	366 (90%)	123 (93%)	243 (88%)	0.200
*Stop treatment with VPA*	147 (36%)	52 (39%)	95 (35%)	0.369
Reason: adverse events	53 (13%)	22 (17%)	31 (11%)	0.138
Reason: interaction of medication*	24 (6%)	7 (5%)	17 (6%)	0.825
Other reasons, no adverse events	70 (17%)	23 (17%)	47 (17%)	0.886
*Adverse events*				
Disturbance of consciousness*	27 (7%)	11 (8%)	16 (6%)	0.397
Thrombocytopenia*	8 (2%)	6 (5%)	2 (1%)	**0.017**
Parkinsonism*	1 (0.2%)	1 (1%)	0	0.326
Elevation of liver enzymes*	7 (2%)	2 (2%)	5 (2%)	1.0
Bleeding*	2 (0.5%)	0	2 (1%)	0.559
Others*	8 (2%)	2 (2%)	6 (2%)	1.0
*In-hospital complications*				
Need for mechanical ventilation	180 (44%)	52 (39%)	128 (47%)	0.156
Episodes of hypoglycemia*	11 (3%)	8 (6%)	3 (1%)	**0.009**
Bleeding with intervention*	20 (5%)	7 (5%)	13 (5%)	0.810
Pancreatic damage*	3 (1%)	1 (1%)	2 (1%)	0.546
*Outcome*				
In-hospital mortality	65 (16%)	26 (20%)	39 (14%)	0.165
In-hospital mortality after recurrent SE*	6/74 (8%)	0/27	6/47 (13%)	0.080
mRS at discharge	4 (3–5)	5 (4–5)	4 (3–5)	**0.004**

Metric variables are described with median and interquartile range, categorial variables in number and percentage. Continuous variables were compared using Mann–Whitney U test. Proportions were compared using Pearson´s Chi2 test or Fisher´s exact test (*). Statistically significant values (*p* < 0.05) are expressed in bold

In another three patients VPA was stopped because of the aggravation of a pre-existing thrombocytopenia

Bold indicates comparison of characteristics between both groups

*STESS* status epilepticus severity score; *GCSE* generalized convulsive status epilepticus; *NCSE* nonconvulsive status epilepticus; *VPA* valproic acid; *SE* status epilepticus; *mRS* modified Rankin scale

### Adverse events during the treatment with VPA and intrahospital complications of patients with or without diabetes

In 14% of the patients, treatment with VPA was stopped due to safety concerns. The most frequent reason for withdrawal of therapy was a disturbance of consciousness (7%).

Univariate analyses revealed no significant difference between the rates of discontinuation in the two groups of patients with or without diabetes (Table 1). The detailed examination of adverse events showed a difference in the rate of thrombocytopenia which was higher in patients with diabetes than in patients without diabetes (5% vs. 1%). The difference remained significant after correction for age and sex in a binary logistic regression model (*p* = 0.015). We also examined the rate of withdrawal of therapy due to concerns about comorbidities or potential adverse events. Those were: thrombocytopenia in the past, aggravation of a known hepatic damage, known osteoporosis, aggravation of hyponatremia, and abuse of alcohol. Therapy with VPA was not stopped more frequently in patients with diabetes because of the concerns mentioned above (*p* = 0.435).

For further evaluation, we also examined the incidence of adverse events in recurrent SE episodes. Out of 408 patients, 61 patients suffered between one and a maximum of four SE episodes treated with IV VPA. In recurrent episodes, there were no significant differences between both groups, either in the rates of discontinuation of therapy or in the rate of specific adverse events including thrombocytopenia (*p* = 0.365, Table 2).

**Table 2:** Adverse events in recurrent SE episodes of the total study cohort and both groups (patients with or without diabetes)

Characteristics	Recurrent SE	Group, diabetes	Group, no diabetes	*p*-value
Episodes	N = 74	N = 27	N = 47	
*Stop treatment with VPA*	19 (26%)	6 (22%)	13 (28%)	0.606
Reason: adverse events*	6 (8%)	3 (11%)	3 (6%)	0.662
Reason: interaction of medication*	5 (7%)	1 (4%)	4 (9%)	0.647
Other reasons, no adverse events*	8 (11%)	2 (7%)	6 (13%)	0.702
*Adverse events*				
Disturbance of consciousness*	2 (3%)	1 (4%)	1 (2%)	1.0
Thrombocytopenia*	1 (1%)	1 (4%)	0	0.365
Parkinsonism*	0	0	0	/
Elevation of liver enzymes*	1 (1%)	0	1 (2%)	1.0
Bleeding*	0	0	0	/
Others*	2 (3%)	1 (4%)	1 (2%)	1.0

In another SE episode VPA was stopped due to an aggravation of a pre-existing thrombocytopenia

Categorial variables were described with number and percentage. Proportions were compared using Pearson´s Chi2 test or Fisher´s exact test (*). Statistically significant values (*p* < 0.05) are expressed in bold

*SE* status epilepticus; *VPA* valproic acid

In the entire cohort and across all SE episodes, a total of 36 hypoglycemic episodes occurred. Out of these, two episodes occurred spontaneously during the therapy with IV VPA, i.e., there was no antidiabetic medication or other known etiology that may predispose to hypoglycemia (such as liver cirrhosis). In one patient without known diabetes, a hypoglycemic episode occurred spontaneously five days after the termination of VPA (Additional file 2).

Within the diabetic group (N = 133), 55 patients were diagnosed with IDDM. Comparison of the two subgroups (patients with or without IDDM) revealed no significant difference in the rate of discontinuation of therapy with VPA or the rate of adverse events (Additional file 3).

### In-hospital mortality and outcome at hospital discharge

Sixty-five of 408 patients (16%) died in hospital. Univariate analysis revealed that patients who died in hospital were treated more often with VPA continuously or with repeated infusions (97%) than patients who survived (88%, *p* = 0.043, Table 3). Potential confounders identified in univariate analyses (age, sex, premorbid mRS, CCI, STESS ≥ 3, potentially fatal etiology, and need for mechanical ventilation) were integrated in a binary logistic regression model. Also, diabetes as covariate was forced into the model. After adjustment for the respective confounders, therapy with IV VPA was not confirmed as an independent risk factor for in-hospital mortality (*p* = 0.079, Additional file 4). In a second step, we repeated the univariate analysis for the group of patients with diabetes. The proportions of patients treated with VPA continuously or in repeated infusions did not differ significantly between the group of patients who died in hospital (100%) and those who survived (91%, *p* = 0.209; Additional file 5).

**Table 3:** Factors associated with in-hospital mortality: total study cohort

Characteristics	In-hospital mortality	Survival of hospital	*p*-value
Patients	N = 65	N = 343	
*Demographics*			
Age	79 (71–87)	72 (60–80)	** < 0.001**
Sex m (male) f (female)	m 21 (32%) f 44 (68%)	m 167 (49%) f 176 (51%)	**0.015**
Premorbid mRS	4 (2 –4)	3 (1–4)	**0.004**
*Comorbidities*			
Charlson Comorbidity Index	4 (3–6)	3 (1–4)	** < 0.001**
Diabetes mellitus	26 (40%)	107 (31%)	0.165
Insulin-dependent diabetes mellitus	7 (11%)	48 (14%)	0.511
*Status epilepticus (SE)*			
STESS ≥ 3	60 (92%)	212 (62%)	** < 0.001**
*Etiology of SE*			
Potentially fatal etiology	44 (68%)	82 (24%)	** < 0.001**
*Treatment with VPA*			
VPA single infusion*	2 (3%)	40 (12%)	**0.037**
VPA repeated or continuous infusion	63 (97%)	303 (88%)	**0.037**
*In-hospital complications*			
Need for mechanical ventilation	37 (57%)	143 (42%)	**0.023**
Episodes of hypoglycemia*	3 (5%)	9 (3%)	0.416
Bleeding with intervention*	2 (3%)	18 (5%)	0.753

Metric variables are described with median and interquartile range, categorial variables in number and percentage. Continuous variables were compared using Mann–Whitney U test. Proportions were compared using Pearson´s Chi2 test or Fisher´s exact test (*). Statistically significant values (*p* < 0.05) are expressed in bold

*mRS* modified Rankin scale; *STESS* status epilepticus severity score; *SE* status epilepticus; *VPA* valproic acid

**Fig. 2:**
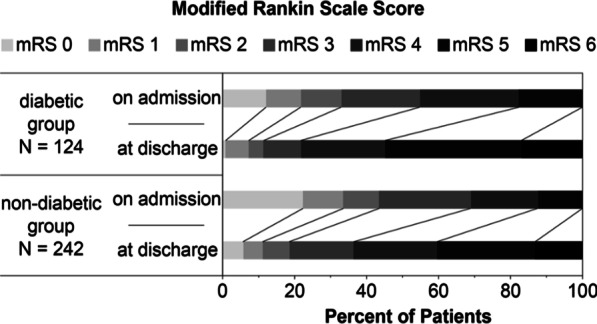
Distribution of mRS scores in both groups patients with or without diabetes. Abbreviations: mRS, modified Rankin Scale

The functional state, as measured by the mRS, deteriorated significantly between the time before the hospital stay and the time at discharge from hospital both in the total cohort (N = 366; *p* < 0.001) and in the two groups (diabetic group: N = 124, *p* < 0.001, non-diabetic group: N = 242; *p* < 0.001). In terms of the proportion of patients with good functional state (mRS 0–3), a significant difference between the diabetic and non-diabetic group could be found in both times: before hospitalization (55% vs. 69%, *p* = 0.008) and at discharge (22% vs. 36%, *p* = 0.004). To further assess the change in functional status during the hospital stay, we compared the differences of the mRS scores between the patients with and without known diabetes. No significant differences could be found (*p* = 0.948). Also, there was no statistically significant interaction between the point in time of mRS determination and group assignment (diabetes vs. non-diabetic; F[1, 364] = 0.026, *p* = 0.873, partial η^2^ < 0.001). The distribution of the premorbid mRS and the functional state at discharge in both groups are illustrated in Fig. 2.

The significant difference of proportions of patients with good functional state before hospitalization and at time of hospital discharge was not reproduced in the separate analysis of the diabetic group: patients with IDDM (N = 50) vs. patients without IDDM (N = 74): premorbid mRS 58% vs. 53%, *p* = 0.586; mRS at time of discharge 26% vs. 19%, *p* = 0.381.

## Discussion

In this retrospective single-center cohort study, we investigated the safety profile of IV VPA in patients with SE focusing on diabetes as a significant and increasingly prevalent comorbidity especially in the elderly population [17, 18]. By including information about this group, the present study complements previous prospective and retrospective trials on the treatment of SE. So far, we have identified only a few studies that considered the impact of comorbidities in the prognosis of SE [4, 19–23].

Our results demonstrate that the rates of discontinuation of VPA therapy do not differ significantly between the diabetic and non-diabetic group. Analysis of individual recurrent SE episodes did not reveal a signal of cumulative toxicity, though the sample size was small. The incidence of adverse events detected in the total cohort was marginally higher than reported in the literature [5]. The difference may be fully explained by the high age of patients (median, 73 years) as advanced age is associated with lower resistance to complications. Regarding safety concerns, a disturbance of consciousness was the most frequent reason leading to discontinuation of treatment. This is in accordance with other studies [9, 24]. Interestingly, the difference in the occurrence of thrombocytopenia remained significant to the disadvantage of the diabetic group even after correction for age and sex. According to previous work, (auto)-immune processes secondary to viral infections [25] or heparin-treatment [26] may play a role in the pathogenesis of thrombocytopenia. A similar mechanism may be operative in the pathogenesis of VPA-induced reduction of platelet counts [27]. Therefore, it seems reasonable to expect that treatment with VPA carries a higher risk of thrombocytopenia with concomitant diabetes. The risk may be enhanced by other predisposing factors (i.e., sepsis). Considering the small number of cases (5% and 2%) the clinical relevance must be assessed individually for each clinical situation.

Long term therapy with VPA is known to cause overweight and hyperglycemia [28–30]. On the other hand, studies in a diabetic rat model or in mice revealed a reduction of insulin resistance and an enhanced insulin- action under short term (10 weeks) or acute treatment with VPA, respectively [31, 32]. In our study, the frequency of hypoglycaemia during VPA treatment and the number of SE episodes were too low to either refute or confirm the hypothesis that VPA is associated with an enhanced risk of hypoglycemia. To clarify this point, prospective studies with a larger cohort of patients are required that include the evaluation of blood glucose levels under therapy with different anticonvulsive drugs.

In several case reports, the treatment with VPA has been associated with acute pancreatitis both in children and adults [e.g., 33, 34]. It is also known that progressive pancreatic beta-cell failure is the key mechanism leading to diabetes mellitus type 1 and 2 [35]. Thus, an enhanced risk of VPA induced pancreatic damage in this predisposed and potentially vulnerable group of patients can be assumed. However, we could not find a significantly different prevalence of acute pancreatic damage in patients with known (insulin-dependent) diabetes in our cohort.

Sixty-five of 408 patients died during hospitalization (16%). This rate is in line with a reported SE related pooled case fatality rate of 14.9% in a meta-analysis (1). While examining VPA as an independent risk factor for in-hospital mortality, we accounted for potential confounders by including variables that were associated with poor outcome in past investigations [3.14–16.36]. In our study, continuous or repeated infusions of VPA were not independently associated with mortality in either the total cohort or the diabetic group. This observation compares well with other investigations in which older age and a more severe etiology of SE rather than the choice of anticonvulsive treatment strategies predicted mortality [3, 37]. Similar to others [3, 38, 39], the potentially fatal etiology was the strongest predictive factor for mortality in different regression models in the present study (OR = 8.177; 95% CI: 3.776–17.7107; *p* < 0.001).

The identification of prognostic factors of SE is grounded by the clear evaluation of outcome parameters. Evaluation of outcome must take into account the premorbid state. The premorbid functional state is likely a prognostic factor itself [40]. In the present study, the diabetic group showed a significantly worse premorbid functional state as indicated by the mRS compared to the non-diabetic group. In view of the known somatic complications of diabetes, which include a greater susceptibility to small vessel disease, this observation seems reasonable. The functional outcome in the short term was significantly worse in the diabetic group, too. Although the in-hospital functional change did not differ between the two groups, these results highlight the vulnerability of patients with diabetes, which is associated with a higher risk of an inacceptable outcome. Therefore, the impact of relevant comorbidities as potential prognostic factors should not only be considered in vascular neurological diseases but also in SE.

To our knowledge, this is the first study that evaluated how the presence of diabetes modulates the safety profile of VPA treatment of SE. An additional strength of the study is the collection of a large set of data comprising overall 482 SE episodes, which exceeds the size of most of the study cohorts in previous work.

The main limitations of the study result from its retrospective and single-center design. Relevant adverse events that may occur in the initial phase of treatment with VPA, such as hypotension and respiratory depression, as observed in prospective studies [9], could not be detected due to methodological reasons. In our study, therapy with VPA was not stopped in any of the episodes with early intubation, which highlights the difficulty in clinical practice to distinguish between SE related or treatment related disturbance of consciousness and respiratory depression.

The adverse events that were attributed to the treatment with VPA were identified only indirectly by the decision to discontinue VPA therapy, which likely has led to an underestimation of the rate of adverse events. On the other hand, this ensures that safety issues are considered, which are deemed clinically sufficiently relevant to justify the discontinuation of therapy.

In the choice of covariates for the binary regression model, not all factors known to be associated with in-hospital mortality could be retrieved from the charts, e.g., duration of SE. In view of this, rather than examining independent risk factors, we focused on whether VPA therapy was associated with in-hospital mortality.

Acute pancreatic damage was not observed in patients without a history of a pancreatic disease. The sample size was too small to investigate the question of whether the prevalence of this exceedingly rare adverse event was higher under VPA therapy in diabetic SE patients. Cases of clinically inapparent, mild pancreatic damage may have been missed, because the presence of pancreatic damage was solely based on the diagnosis of the treating clinicians, and full pancreatic testing was not performed in the study population. However, even if we cannot rule out that VPA may increase the risk for pancreatic damage in diabetic patients, its clinical relevance must be weighed carefully against the clinical risk associated with not stopping SE or stopping it too late, and the limited number of available therapies.

The present study included patients with all types of diabetes. This condition potentially leads to an underestimation of relevant adverse events because the subgroup of patients with IDDM was small. The small group size may prevent the recognition of significant differences due to the lack of statistical power. With regard to the small incidence of patients with SE plus IDDM (in this study 13% of the IV VPA cohort), our approach may cover reality to the best possible degree.

The German Instruction for Use prohibits the IV treatment of VPA in patients with IDDM in all marketed preparations [41, 42]. Based on our results, this restriction does not seem justified. Prolonged SE is associated with a significant personal and economic burden [43, 44] highlighting the importance of effective treatment strategies. The present study provides no evidence that patients with IDDM and SE are at higher risk of significant treatment related complications when receiving IV treatment with VPA.

## Conclusions

In the present study, tolerability of treatment with IV VPA was similar in patients with or without diabetes. The observation of a higher incidence of drug associated thrombocytopenia in patients with diabetes should prompt increased awareness of this complication in the clinical setting. The study suggests that diabetes should be considered as a relevant comorbidity that, alone or as an indicator of the presence of other factors, can signal a potentially increased risk of a poor outcome after SE.

## Supplementary Information

**Additional file 1** Details about the therapy with valproic acid. VPA was given either continuously via perfusor or with repeated infusions with a maximum of five times per day. Only patients who received the maintenance dosage (intravenous) for at least 24 hours were considered. 33 patients received VPA as oral therapy. Proportions of patients were compared using Pearson´s Chi2 test or Fisher´s exact test (*). Abbreviations: VPA, valproic acid.

**Additional file 2** Characteristics of hypoglycemic episodes, separated by patients. Out of 15 patients 12 patients had a known diabetes mellitus, 6 had an insulin-dependent diabetes mellitus. Out of 36 hypoglycemic episodes 29 episodes occurred under treatment with antidiabetics. One hypoglycemic episode occurred spontaneously under treatment with intravenous valproic acid, two episodes occurred independently from valproic acid or antidiabetic treatment. Abbreviations: BG, blood glucose, values in mmol/L; DM, diabetes mellitus; IDDM insulin-dependent diabetes mellitus; IV, intravenous; VPA, valproic acid; SC, subcutaneously. x means yes; / means no.

**Additional file 3** Characteristics of patients with diabetes and both subgroups (insulin-dependent and non-insulin-dependent diabetes) Metric variables are described with median and interquartile range, categorial variables in number and percentage. Continuous variables were compared using Mann-Whitney U test. Proportions were compared using Pearson´s Chi2 test or Fisher´s exact test (*). Statistically significant values (*p*<0.05) are expressed in bold. Abbreviations: DM, diabetes mellitus; IDDM, insulin-dependent diabetes mellitus; NIDDM non-insulin-dependent diabetes mellitus; STESS, Status Epilepticus Severity Score; VPA, valproic acid; SE, status epilepticus; mRS, modified Rankin Scale.

**Additional file 4** Binary regression model for in-hospital mortality Statistically significant values (*p*<0.05) are expressed in bold. Abbreviations: OR, Odds Ratio; CI, Confidence Interval; mRS, modified Rankin Scale; STESS, Status Epilepticus Severity Score; VPA, valproic acid.

**Additional file 5** Factors associated with in-hospital mortality: group of patients with diabetes Metric variables are described with median and interquartile range, categorial variables in number and percentage. Continuous variables were compared using Mann-Whitney U test. Proportions were compared using Pearson´s Chi2 test or Fisher´s exact test (*). Statistically significant values (*p*<0.05) are expressed in bold. Abbreviations: mRS, modified Rankin Scale; STESS, Status Epilepticus Severity Score; SE, status epilepticus; VPA, valproic acid.

## Data Availability

The datasets analysed during the current study are available from the corresponding author on reasonable request.

## Abbreviations

CCI: Charlson comorbidity index
ESETT: Established status epilepticus treatment trial
IDDM: Insulin-dependent diabetes mellitus
IV: Intravenous
mRS: Modified Rankin scale
NIDDM: Non-insulin-dependent diabetes mellitus
SE: Status epilepticus
STESS: Status epilepticus severity score
VPA: Valproic acid
