# Supplementary material for: Does diabetes mellitus affect the safety profile of valproic acid for the treatment of status epilepticus? A retrospective cohort study
Source: Neurol Res Pract. 2022 Oct 24;4:52. doi: 10.1186/s42466-022-00212-w (PMC9590127; doi:10.1186/s42466-022-00212-w)
Supplement: Supplementary file 1 — Additional file 1 Details about the therapy with valproic acid. VPA was given either continuously via perfusor or with repeated infusions with a maximum of five times per day. Only patients who received the maintenance dosage (intravenous) for at least 24 hours were considered. 33 patients received VPA as oral therapy. Proportions of patients were compared using Pearson´s Chi2 test or Fisher´s exact test (*). Abbreviations: VPA, valproic acid. [file 42466_2022_212_MOESM1_ESM.pdf]

Additional File 1- Details about the therapy with valproic acid

Data about the time when therapy with VPA was started were available in N= 396 patients. In 80 % of cases, therapy with VPA started on the same day that SE was diagnosed, and in 98 % of the patients, VPA treatment started within the first three days. The proportion of patients in whom treatment with VPA was started on the first day or within the first three days did not differ between the diabetic and non-diabetic group (first day: 81 % vs 80 %, p = 0.776; within three days: 96 % vs. 99 % %, p= 0.131). Data about the loading dosage of VPA could be obtained in N= 401 patients. Out of this cohort, treatment was initiated in 78 % with an IV loading dose ranging between 300 and 1500 mg; the majority of patients (61 %) received 900 mg VPA. The maintenance dosage of patients who received IV VPA for at least 24 hours ranged between 1150 and 4800 mg (maximum dosage per day); further details are listed in the following Table.

**Dosage VPA**	**Total cohort**	**Group, diabetes**	**Group, no diabetes**	**p-value**
(maximum, per day)	N= 306	N= 109	N= 197	
Between 1000 and 1999 mg	40 (13 %)	17 (16 %)	23 (12 %)	0.330
Between 2000 and 2999 mg	217 (71 %)	75 (69 %)	142 (72 %)	0.546
Between 3000 and 3999 mg	45 (15 %)	16 (15 %)	29 (15 %)	0.992
4000 and more	4 (1 %)	1 (1 %)	3 (2 %)	1.0

Table Additional File 1: Details of the intravenous maintenance dosage of valproic acid per day
VPA was given either continuously via perfusor or with repeated infusions with a maximum of five times per day. Only patients who received the maintenance dosage (intravenous) for at least 24 hours were considered. 33 patients received VPA as oral therapy. Proportions of patients were compared using Pearson´s Chi2 test or Fisher´s exact test (*). Abbreviations: VPA, valproic acid.
