# Supplementary material for: Does diabetes mellitus affect the safety profile of valproic acid for the treatment of status epilepticus? A retrospective cohort study
Source: Neurol Res Pract. 2022 Oct 24;4:52. doi: 10.1186/s42466-022-00212-w (PMC9590127; doi:10.1186/s42466-022-00212-w)
Supplement: Supplementary file 2 — Additional file 2 Characteristics of hypoglycemic episodes, separated by patients. Out of 15 patients 12 patients had a known diabetes mellitus, 6 had an insulin-dependent diabetes mellitus. Out of 36 hypoglycemic episodes 29 episodes occurred under treatment with antidiabetics. One hypoglycemic episode occurred spontaneously under treatment with intravenous valproic acid, two episodes occurred independently from valproic acid or antidiabetic treatment. Abbreviations: BG, blood glucose, values in mmol/L; DM, diabetes mellitus; IDDM insulin-dependent diabetes mellitus; IV, intravenous; VPA, valproic acid; SC, subcutaneously. x means yes; / means no. [file 42466_2022_212_MOESM2_ESM.pdf]

Patient	Episode(s)	BG level	DM	IDDM	IV VPA	After stop of VPA (duration)	Antidiabetic therapy	Other potential etiologies
**1**	1	2.6	x	/	x	/	oral (sulfonylurea)	/
**2**	8	1.6	x	x	1	7 (minimum 13.5 hours)	all episodes under insulin IV	/
**3**	1	2.8	/	/	/	x (4 hours)	/	liver cirrhosis
**4**	2	3.1	x	/	1	/	/	/
**5**	2	1.5	x	/	/	1 (8.5 hours)	1 under insulin IV	abuse of alcohol
**6**	1	3.2	x	/	/	/	oral (sitagliptin, metformin)	/
**7**	1	3.2	/	/	/	x (5 days)	/	/
**8**	5	1.3	x	x	/	/	SC (insulin glargin/ insulin glulisine)	/
**9**	1	3.1	x	x	x	/	insulin IV	/
**10**	7	1.2	x	x	1	/	all episodes under insulin IV	/
**11**	1	2.6	x	x	x	/	/	/
**12**	2	2.1	/	/	/	/	/	phenytoin intoxication
**13**	1	1.8	x	/	/	/	oral (sitagliptin)	/
**14**	3	2.4	x	x	/	/	all episodes under insulin IV	septic shock

Table Additional File 2: Characteristics of hypoglycemic episodes, separated by patients
Considered were all SE-episodes (N = 482). Out of 14 patients, 11 patients had a known diabetes mellitus, 6 had an insulin-dependent diabetes mellitus. Out of 36 hypoglycemic episodes, 28 episodes occurred under treatment with antidiabetics. Two hypoglycemic episodes occurred spontaneously under treatment with intravenous valproic acid, one episode occurred independently from valproic acid or antidiabetic treatment. Abbreviations: BG, blood glucose, values in mmol/L; DM, diabetes mellitus; IDDM insulin-dependent diabetes mellitus; IV, intravenous; VPA, valproic acid; SC, subcutaneously. x means yes; / means no
