# Supplementary material for: Does diabetes mellitus affect the safety profile of valproic acid for the treatment of status epilepticus? A retrospective cohort study
Source: Neurol Res Pract. 2022 Oct 24;4:52. doi: 10.1186/s42466-022-00212-w (PMC9590127; doi:10.1186/s42466-022-00212-w)
Supplement: Supplementary file 3 — Additional file 3 Characteristics of patients with diabetes and both subgroups (insulin-dependent and non-insulin-dependent diabetes) Metric variables are described with median and interquartile range, categorial variables in number and percentage. Continuous variables were compared using Mann-Whitney U test. Proportions were compared using Pearson´s Chi2 test or Fisher´s exact test (*). Statistically significant values (p<0.05) are expressed in bold. Abbreviations: DM, diabetes mellitus; IDDM, insulin-dependent diabetes mellitus; NIDDM non-insulin-dependent diabetes mellitus; STESS, Status Epilepticus Severity Score; VPA, valproic acid; SE, status epilepticus; mRS, modified Rankin Scale. [file 42466_2022_212_MOESM3_ESM.pdf]

Characteristics	Group, diabetes	Subgroup, IDDM	Subgroup, NIDDM	p-value
Patients	N= 133	N= 55	N= 77	
**Demographics**				
Age	75 (70 -- 81)	73 (68 -- 78)	76 (71 -- 83)	**0.037**
Sex: m (male) f (female)	m 51 (38 %), f 82 (62 %)	m 23 (42 %), f 32 (58 %)	m 28 (36 %), f 49 (64 %)	0.526
**Comorbidities**				
Charlson Comorbidity Index	4 (3 -- 6)	4 (3 -- 6)	4 (3 -- 6)	0.348
Active abuse of alcohol*	10 (8 %)	5 (9 %)	5 (7 %)	0.741
**Status epilepticus**				
STESS ≥ 3	106 (80 %)	42 (76 %)	63 (82 %)	0.444
History of seizures	48 (36 %)	20 (36 %)	28 (36 %)	1.0
**Etiology of SE**				
Potentially fatal etiology	47 (35 %)	21 (38 %)	25 (33 %)	0.497
**Treatment with VPA**				
VPA single infusion	10 (8 %)	6 (11 %)	4 (5 %)	0.318
VPA repeated or continuous infusion	123 (93 %)	49 (89 %)	73 (95 %)	0.221
**Stop treatment with VPA**	52 (39 %)	26 (47 %)	26 (34 %)	0.117
Reason: adverse events	22 (17 %)	12 (22 %)	10 (13 %)	0.180
Reason: interaction of medication*	7 (5 %)	5 (9 %)	2 (3 %)	0.127
Other reasons, no adverse events	23 (17 %)	9 (16 %)	14 (18 %)	0.786
**Adverse events**				
Disturbance of consciousness*	11 (8 %)	5 (9 %)	6 (8 %)	1.0
Thrombocytopenia*	6 (5 %)	3 (6 %)	3 (4 %)	0.693
Parkinsonism*	1 (1 %)	0	1 (1 %)	1.0
Elevation of liver enzymes*	2 (2 %)	2 (4 %)	0	0.172
Bleeding*	0	0	0	/
Others*	2 (3 %)	2 (4 %)	0	0.172
**In-hospital complications**				
Need for mechanical ventilation	52 (39 %)	26 (47 %)	26 (34 %)	0.149
Episodes of hypoglycemia*	8 (6 %)	5 (9 %)	3 (4 %)	0.219
Bleeding with intervention*	7 (5 %)	5 (9 %)	2 (3 %)	0.127
Pancreatic damage*	1 (1 %)	1 (2 %)	0	0.417
**Outcome**				
In-hospital mortality	26 (20 %)	7 (13 %)	18 (23 %)	0.124
mRS at discharge	5 (4 -- 5)	5 (4 -- 5)	5 (4 -- 5)	0.361

Table Additional File 3: Characteristics of patients with diabetes and both subgroups (insulin-dependent and non-insulin-dependent diabetes)
Metric variables are described with median and interquartile range, categorial variables in number and percentage. Continuous variables were compared using Mann-Whitney U test. Proportions were compared using Pearson´s Chi2 test or Fisher´s exact test (*). Statistically significant values ($p<0.05$) are expressed in bold. Abbreviations: IDDM, insulin-dependent diabetes mellitus; NIDDM non-insulin- dependent diabetes mellitus; STESS, Status Epilepticus Severity Score; VPA, valproic acid; SE, status epilepticus; mRS, modified Rankin Scale
