# Supplementary material for: Does diabetes mellitus affect the safety profile of valproic acid for the treatment of status epilepticus? A retrospective cohort study
Source: Neurol Res Pract. 2022 Oct 24;4:52. doi: 10.1186/s42466-022-00212-w (PMC9590127; doi:10.1186/s42466-022-00212-w)
Supplement: Supplementary file 4 — Additional file 4 Binary regression model for in-hospital mortality Statistically significant values (p<0.05) are expressed in bold. Abbreviations: OR, Odds Ratio; CI, Confidence Interval; mRS, modified Rankin Scale; STESS, Status Epilepticus Severity Score; VPA, valproic acid. [file 42466_2022_212_MOESM4_ESM.pdf]

Covariate	OR	95 % CI	p-value
Age	1.073	1.032 - 1.116	**<0.001**
Sex	1.921	0.891 - 4.141	0.096
Premorbid mRS	1.272	1.013 - 1.599	**0.039**
Charlson Comorbidity Index	1.237	1.028 - 1.489	**0.024**
Diabetes mellitus	0.550	0.252 - 1.204	0.135
STESS ≥ 3	3.270	0.982 - 10.895	0.054
Potentially fatal etiology	8.177	3.776 - 17.707	**<0.001**
VPA repeated or continuous infusion	7.306	0.797 - 66.977	0.079
Need for mechanical ventilation	1.744	0.825 - 3.686	0.145

Table Additional File 4: Binary regression model for in-hospital mortality
Statistically significant values ($p<0.05$) are expressed in bold. Abbreviations: OR, Odds Ratio; CI, Confidence Interval; mRS, modified Rankin Scale; STESS, Status Epilepticus Severity Score; VPA, valproic acid
