# Supplementary material for: Does diabetes mellitus affect the safety profile of valproic acid for the treatment of status epilepticus? A retrospective cohort study
Source: Neurol Res Pract. 2022 Oct 24;4:52. doi: 10.1186/s42466-022-00212-w (PMC9590127; doi:10.1186/s42466-022-00212-w)
Supplement: Supplementary file 5 — Additional file 5 Factors associated with in-hospital mortality: group of patients with diabetes Metric variables are described with median and interquartile range, categorial variables in number and percentage. Continuous variables were compared using Mann-Whitney U test. Proportions were compared using Pearson´s Chi2 test or Fisher´s exact test (*). Statistically significant values (p<0.05) are expressed in bold. Abbreviations: mRS, modified Rankin Scale; STESS, Status Epilepticus Severity Score; SE, status epilepticus; VPA, valproic acid. [file 42466_2022_212_MOESM5_ESM.pdf]

Characteristics	In-hospital mortality	Survival of hospital	p-value
Patients	N= 26	N= 107	
**Demographics**			
Age	77 (72 -- 87)	75 (68 -- 80)	0,080
Sex m (male) f (female)	m 8 (31 %) f 18 (69 %)	m 43 (40 %) f 64 (60 %)	0.501
Premorbid mRS	4 (2 -- 4)	3 (2 -- 4)	0.582
**Comorbidities**			
Charlson Comorbidity Index	5 (4 -- 7)	4 (3 -- 5)	**0.004**
Insulin-dependent diabetes mellitus	7 (27 %)	48 (45 %)	0.176
**Status epilepticus (SE)**			
STESS ≥ 3	26 (100 %)	80 (75 %)	**0.005**
**Etiology of SE**			
Potentially fatal etiology	17 (65 %)	30 (28 %)	**<0.001**
**Treatment with VPA**			
VPA repeated or continuous infusion	26 (100 %)	97 (91 %)	0.209
**In-hospital complications**			
Need for mechanical ventilation	15 (58 %)	37 (35 %)	**0.043**
Episodes of hypoglycemia	3 (12 %)	6 (6 %)	0.377
Bleeding with intervention*	1 (4 %)	6 (6 %)	1.0
Pancreatic damage*	1 (4 %)	0	0.195

Table Additional File 5: Factors associated with in-hospital mortality: group of patients with diabetes
Metric variables are described with median and interquartile range, categorial variables in number and percentage. Continuous variables were compared using Mann-Whitney U test. Proportions were compared using Pearson´s Chi2 test or Fisher´s exact test (*). Statistically significant values ($p<0.05$) are expressed in bold. Abbreviations: mRS, modified Rankin Scale; STESS, Status Epilepticus Severity Score; SE, status epilepticus; VPA, valproic acid
